# The Role of Insulin Resistance in the Development of Complications after Coronary Artery Bypass Grafting in Patients with Coronary Artery Disease

**DOI:** 10.3390/biomedicines11112977

**Published:** 2023-11-05

**Authors:** Alexey N. Sumin, Natalia A. Bezdenezhnykh, Andrey V. Bezdenezhnykh, Anastasiya V. Osokina, Anastasiya A. Kuzmina, Anna V. Sinitskaya, Olga L. Barbarash

**Affiliations:** Federal State Budgetary Institution “Research Institute for Complex Issues of Cardiovascular Diseases”, Sosnovy Blvd. 6, Kemerovo 650002, Russia; an_sumin@mail.ru (A.N.S.); andrew22014@mail.ru (A.V.B.); av.osokina80@yandex.ru (A.V.O.); stusha76@mail.ru (A.A.K.); annacepokina@mail.ru (A.V.S.); olb61@mail.ru (O.L.B.)

**Keywords:** coronary bypass grafting, carbohydrate metabolism disorders, postoperative complications, insulin resistance indices, free fatty acids

## Abstract

The aim of the study was to investigate the effect of carbohydrate metabolism disorders and insulin resistance indices on the immediate results of coronary artery bypass grafting (CABG). Method. Patients with coronary artery disease who underwent CABG (*n* = 383) were examined to determine glycemic status, free fatty acid and fasting insulin levels, and insulin resistance indices (Homeostasis Model Assessment of Insulin Resistance (HOMA-IR), McAuley index, Quantitative Insulin Sensitivity Check Index (QUICKI), Revised-QUICKI). Patients were assessed for the development of perioperative complications and their length of stay in the hospital. Two groups were formed: group 1, patients with a combined endpoint (CEP, any complication and/or duration of hospital stay >10 days), *n* = 291; and group 2 (*n* = 92) without a CEP. Perioperative characteristics were analyzed, and predictors of hospital complications and prolonged hospital stay were evaluated. Results. Patients in the CEP group were older, and there were more women among them (*p* = 0.003). Additionally, in this group, there were more patients with diabetes mellitus (37.5% vs 17.4%, *p* < 0.001), obesity (*p* < 0.001), and a higher percentage of combined operations (*p* = 0.007). In the group with a CEP, the levels of glucose (*p* = 0.031), glycated hemoglobin (*p* = 0.009), and free fatty acids (*p* = 0.007) and the Revised-QUICKI (*p* = 0.020) were higher than in the group without complications. In a regression analysis, the independent predictors of complications were combined operations (*p* = 0.016) and the predictors of a long hospital stay (>14 days) were female gender, the left atrium size, and diabetes mellitus (*p* < 0.001). The predictors of a composite endpoint included female gender, age, the left atrium size, and free fatty acid levels (*p* < 0.001). Conclusions: In the group with in-hospital complications after CABG, not only was the presence of diabetes mellitus more often detected, but there were also higher levels of free fatty acids and a higher Revised-QUICKI. Therefore, additional assessments of insulin resistance and free fatty acid levels are advisable in patients before CABG.

## 1. Introduction

At present, the high prevalence of diabetes mellitus (DM) among patients with coronary artery disease (CAD) in general [1] and among patients undergoing coronary artery bypass grafting (CABG) [2] is beyond doubt. Moreover, the frequency of DM among these patients continues to grow along with an increase in the prevalence of DM among the general population [3]. Since the presence of concomitant DM not only affects the choice of the myocardial revascularization strategy [4,5], but also the prognosis after such interventions [6], attention is now being paid not only to the presence of DM, but also to the degree of glycemic control [7,8,9]. In addition, since even the presence of prediabetes can affect the prognosis in cardiac patients [10], active screening of early carbohydrate metabolism disorders before coronary interventions is justified [11,12,13]. Moreover, some researchers go further, evaluating the indexes of insulin resistance in patients before cardiac surgery [14,15]. A rationale for such studies is the fact that insulin resistance indices are associated with the severity of coronary artery disease [16] and the progression of coronary atherosclerosis [17]. However, so far, only a few papers have been devoted to the study of insulin resistance indices during cardiac surgery. Accordingly, the purpose of this study was to investigate the influence of carbohydrate metabolism disorders and insulin resistance in the development of complications of coronary artery bypass grafting in patients with coronary artery disease.

## 2. Materials and Methods

### 2.1. Study Design

Consecutive patients who underwent elective CABG in the Department of Cardiovascular Surgery of the Kemerovo Research Institute clinic from 22 March 2011 to 22 October 2012 were included in an observational registry cross-sectional study. The study was performed in accordance with the Good Clinical Practice standards and the principles of the Declaration of Helsinki. The study protocol was approved (date of approval: 7 September 2011) by the Local Ethical Committee of Research Institute for Complex Issues of Cardiovascular Diseases (Protocol No. 20110907). Informed consent was obtained from all subjects involved in the study.

**Figure 1:**
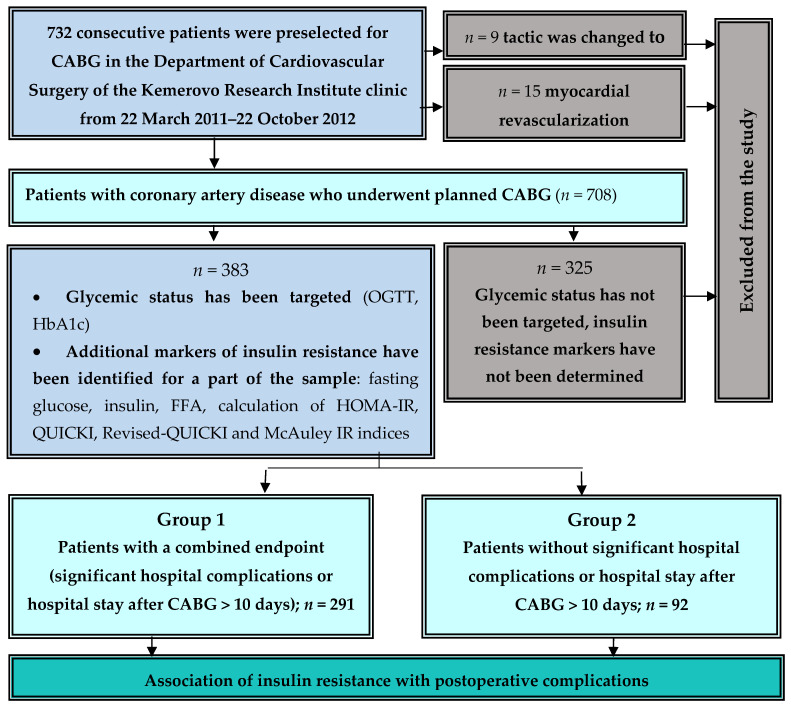
Flowchart of patient selection. **Notes:** CABG—coronary artery bypass grafting, OGTT—oral glucose tolerance test, FFAs—free fatty acids, HOMA-IR—Homeostasis Model Assessment of Insulin Resistance, HbA1c—glycated hemoglobin, QUICKI—Quantitative Insulin Sensitivity Check Index.

The study inclusion scheme is shown in Figure 1. A total of 732 consecutive patients were preselected for CABG; in 9 of them, the tactics of revascularization were changed to endovascular intervention and 15 patients were denied surgical treatment; they were excluded from the study. Of the 708 registered patients undergoing CABG, free fatty acids (FFAs) and fasting insulin were determined in 383 consecutive patients and the following calculated indices of insulin resistance were determined: Homeostasis Assessment Model of Insulin Resistance (HOMA-IR), McAuley insulin resistance index, QUICKI (Quantitative Control Index of Insulin Sensitivity) and Revised-QUICKI.

All of these 383 patients were examined for glycemic status before CABG. The remaining 325 patients for whom no additional laboratory markers were evaluated were excluded from the analysis.

The combined endpoint (CEP) was determined as the onset of one of the significant hospital complications or hospital stay after CABG > 10 days. Patients were divided into 2 groups: group 1 consisted of patients with CEP (*n* = 291) and group 2 contained patients who did not have complications or require a long-term hospital stay (*n* = 92) (Figure 1). In patients of these groups, perioperative characteristics were analyzed and the predictors of hospital complications and long-term hospital stay were evaluated.

### 2.2. Data Collection

In the studied patients, the data of anamnesis, routine laboratory examinations, echocardiography (extended protocol), coronary angiography, ultrasound and angiographic examination of the aorta, brachiocephalic and peripheral arterial pools, and the frequency of postoperative complications were analyzed.

### 2.3. Postoperative Complications

As significant postoperative complications of CABG, myocardial infarction (MI) (intra- and postoperative); heart failure requiring inotropic support; atrial fibrillation; stroke; acute kidney injury or the need for renal replacement therapy; multiple organ failure; respiratory failure; pneumonia; hydrothorax requiring puncture; various complications from the sternal wound; diastasis of the sternum; mediastinitis; bleeding; and remediastinotomy for bleeding were taken into account. We also analyzed all deaths after CABG during the hospital stay.

### 2.4. Assessment of Glycemic Status and Insulin Resistance Indices

Diagnosis of carbohydrate metabolism disorders was carried out in accordance with the current recommendations [18] and was described in detail by us previously [15]. Determination of free fatty acids in blood serum was carried out using the spectrophotometric method, using standard test systems from “Thermo Fisher Scientific” (Helsinki, Finland) on a Konelab 30i automatic biochemical analyzer from the same company.

The HOMA-IR index was calculated using the formula: HOMA-IR = glucose × insulin/22.5. As insulin resistance increases, the HOMA-IR index increases accordingly. Glucose in this equation was measured in mmol/L, insulin in μU/mL.

The following equations were used to calculate the QUICKI and the Revised-QUICKI:

QUICKI = 1/log(glucose) + log(insulin)(1)

Revised-QUICKI = 1/(log(glucose) + log(insulin) + log(FFA))(2)

Glucose in this equation was measured in mg/dL, insulin in mIU/mL, and free fatty acids in mmol/L.

The McAuley index was calculated using the equation [19]:

McAuley = exp {2.63 − 0.28 ln [insulin (IU/mL)] − 0.31 ln [triglycerides (mmol/L)]}(3)

The lower the QUICKI, Revised-QUICKI and McAuley index values, the higher the degree of insulin resistance. Reference values for most calculated indices of insulin resistance have not been determined. Laboratory indicators for calculating all the described IR indices were determined in fasting blood serum before surgery [19].

### 2.5. Description of the Coronary Bypass Procedure

Coronary artery bypass grafting was performed under conditions of a cardiopulmonary bypass (CPB) and cardioplegia, on a beating heart under conditions of a parallel CPB, and on a beating heart according to the OPCAB method (off-pump coronary artery bypass) without using a CPB. Operations under conditions of a parallel CPB were performed in the systemic normothermia mode using Octopus vacuum stabilization systems. The decision to perform coronary artery bypass grafting was made by a multidisciplinary team, taking into account the current national and international recommendations at that time. Perioperative management of glycemia was carried out in accordance with the current national recommendations at that time.

### 2.6. Statistical Analyses

SPSS 26.0 software was used for statistical calculations. Since the distribution of all quantitative characteristics differed from normal (when using the Shapiro–Wilk test), medians, upper and lower quartiles (25th and 75th percentiles) were used to describe them. The groups were compared using the Mann–Whitney test and the χ^2^ (chi-square) test; in the case of a small number of observations, Fisher’s exact test with Yates’ correction was used.

We used binary logistic regression to identify factors independently associated with in-hospital CABG outcomes. Regression models were built for four outcomes: (1) for the development of any significant hospital complication; (2) for a hospital stay after CABG > 10 days; (3) for a hospital stay after CABG > 14 days; (4) for the combined endpoint (significant complications or a hospital stay after CABG > 10 days). The binary logistic regression models included variables in which the groups with and without perioperative complications differed (clinical and anamnestic data, laboratory parameters, echocardiography indicators, characteristics of the operating period—see Table 1 and Table 2). Differences were considered significant at (*p*) less than 0.05.

## 3. Results

In the group with a complicated postoperative period, the median age was higher and the proportion of women was 30% vs 14.1% in patients with complications (*p* = 0.003). Patients with complications had a higher percentage of carbohydrate metabolism disorders due to type 2 diabetes (Table 1). In addition, patients with complications had a higher median body mass index and a higher level of obesity (*p* < 0.001 in both cases). Patients of the groups did not differ in cardiovascular history; group 1 had a higher operational risk according to the EuroSCORE II scale (*p* = 0.002).

In the group with complications, operations were more often performed under cardiopulmonary bypass (CPB) conditions; the median CPB duration and the total duration of the operation were significantly higher (*p* = 0.002 and *p* = 0.006, respectively). It is noteworthy that almost all combined operations were performed in the group with complications. In the group with complications, the median of distal anastomoses was higher (*p* < 0.001). When evaluating preoperative drug therapy, there were no significant differences, with the exception of antihyperglycemic therapy; patients with complications were significantly more likely to receive sulfonylurea drugs and insulin before surgery (*p* = 0.004 and *p* = 0.002, respectively).

Patients in group 1 had higher median triglycerides; there were no differences in other routine lipid profile parameters. The median fasting glucose and the glycated hemoglobin were also higher in the group with complications (*p* = 0.031 and *p* = 0.009, respectively). At the same time, free fatty acids and the Revised-QUICKI calculated on their basis were significantly higher in the group with complications (*p* = 0.007 and *p* = 0.020, respectively). At the same time, the median insulin, QUICKI and McAuley IR indices did not differ in the groups. HOMA-IR showed a trend towards a higher value in the group with complication, which did not reach statistical significance (*p* = 0.083).

In the group with complications, the median size of the left atrium and the mass of the left ventricular myocardium were larger, the rest of the echocardiographic parameters were comparable (Table 2). Coronary angiography scores were comparable across groups (Table 2).

**Figure 2:**
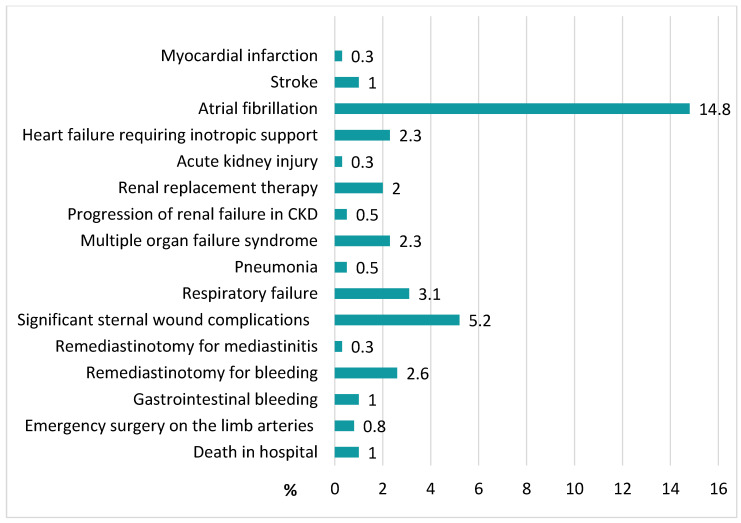
Postoperative hospital complications (*n* = 383).

Postoperative hospital complications are shown in Figure 2. 4 of the patients (1.0%) who died in the hospital, all deaths were due to a cardiovascular cause. In addition, one patient had a perioperative non-fatal myocardial infarction (0.3%) and four patients had non-fatal ischemic strokes (1.0%). Heart failure developed in 2.3% of patients.

Direct selection binary logistic regression (likelihood ratio) was used to identify predictors of a long hospital stay or significant hospital complications in four models (Table 3). The composite endpoint was hospitalization longer than 10 days or a significant hospital complication. All hospital complications described in Section 2 were considered significant.

According to the binary regression results, only combined operations became predictors of significant complications; other factors did not show a connection with this outcome (Table 3). The regression model was statistically significant, χ^2^(2) = 5.83, *p* = 0.016. The model explained 5.6% of the outcome variance (Nagelkerke R2 was 0.056) and correctly classified 77.8% of cases (Supplementary Table S3).

Direct selection binary logistic regression (likelihood ratio) was used to identify predictors of a long hospital stay or significant hospital complications. The composite endpoint was more than 10 days of hospitalization or a significant hospital complication. All hospital complications described in Section 2 were considered significant.

Only combined operations were predictors of significant complications based on the result of binary regression; other factors did not show a connection with this outcome (Table 3). The regression model was statistically significant, χ^2^(2) = 5.83, *p* = 0.016. The model explained 5.6% of the outcome variance (Nagelkerke R2 was 0.056) and correctly classified 77.8% of cases (Supplementary Tables S1–S3).

Gender, increased left atrial size, and LV end-diastolic volume predicted a hospital stay after CABG of >10 days (Table 3). The B value for the male gender is negative (B = −1.982); thus, a male gender reduced the probability of the event occurring by 198.2%. In this model, χ^2^(2) was 38.365 (*p* < 0.001), Nagelkerke R2 was 0.33, and the model correctly classified 77.2% of cases (Supplementary Tables S1–S3).

When analyzing the predictors of a hospital stay after CABG of >14 days, in addition to the same factors (gender, left atrium), type 2 diabetes mellitus showed an influence; the probability of long-term hospitalization in its presence increased by 119.6% (Table 3). The regression model was statistically significant, χ^2^(2) = 20.881, *p* < 0.001. The model explained 17.8% of the outcome variance (Nagelkerke R2 was 0.178) and correctly classified 72.2% of cases (Supplementary Tables S1–S3).

The predictors of combined disease (significant complications or a hospital stay after CABG of >10 days) were gender, age, left atrium, and free fatty acids. With an increase in FFAs by 1 mmol l, the probability of an end point increased by 255.9%, and with an increase in the LA of 1 cm, this probability increased by 219.5%; each year of age added 6.9% to the probability of an index event. Although the value of Nagelkerke’s R2 was relatively low at 0.336, explaining 33.6% of the combined variance in the results, the model correctly classified 78.5% of the cases. Also, this model showed a high statistical significance (χ^2^(2) = 38.337, *p* < 0.001) (Supplementary Tables S1–S3).

At the same time, other biomarkers (fasting levels of glucose, insulin, lipids, and HOMA, QUICKI, Revised-QUICKI, McAuley indices) did not show any association with complications after CABG.

## 4. Discussion

The present study showed that in the group with the development of perioperative complications and/or a long stay in the hospital, carbohydrate metabolism disorders such as diabetes mellitus and carbohydrate metabolism disorders in general, as well as a decrease in the insulin resistance index (Revised-QUICKI) and an increase in the concentration of free fatty acids, were significantly more often detected. It was also found that among the independent predictors of the presence of a combined endpoint (major adverse cardiovascular events + duration of hospitalization of more than 10 days), along with gender, age and size of the left atrium, there was also the level of free fatty acids before surgery. In turn, diabetes mellitus was one of the independent predictors of a length of stay in hospital of more than 14 days.

Indeed, it is known that perioperative factors (surgical trauma, cardiopulmonary bypass, etc.) can lead to insulin resistance and impaired glucose utilization, thereby causing stress hyperglycemia. Insulin resistance persists up to 24 h after surgery [20]. The exact mechanism of insulin resistance during a cardiopulmonary bypass remains poorly understood. For example, the experiment shows that a decrease in the expression of the *p*-AMP-activated protein kinase protein after myocardial ischemia–reperfusion reduces the expression of the GLUT-4 protein (insulin-dependent glucose transport protein), which leads to an impaired glucose uptake and utilization by the myocardium [21]. This mechanism may be one of the causes of myocardial resistance to insulin and may lead to the development of additional myocardial damage during ischemia–reperfusion. The increase in the level of the HOMA-IR index in the perioperative period was more pronounced in patients with initially elevated fasting glucose levels [20]. It can be assumed that an initially increased IR also contributes to a more pronounced increase in IR during surgery, but there are no such data in patients undergoing CABG. The clinical significance of an increase in the level of IR is still not fully understood. Thus, among these patients, an increase in brain damage markers (neuron-specific enolase and S100B) is detected, but there are no clinical signs of brain damage [20]. This is consistent with the conflicting data of our studies. In a study in 2021, we showed that the Disse IR index was an independent factor in the development of postoperative complications [15]. At the same time, in the present study, one of the IR indices—the Revised-QUICKI—turned out to be higher in the group with a combined endpoint (complications + duration of hospitalization), but did not have an independent influence on the development of this combined endpoint.

Regarding FFAs, it was previously shown that during a follow-up of three years in a cohort of patients with the angiographically confirmed presence of CAD, elevated FFAs were an independent predictor of cardiovascular events, which included all-cause mortality, myocardial infarction, ischemic stroke and coronary revascularization. In patients with the highest FFA values (fourth quartile), the risk of developing all-cause mortality was 4.11 times higher than in patients with the lowest FFA values (first quartile) [22]. In critically ill patients, patients with high FFA levels were significantly older, more likely to have diabetes and glycemia and have higher concentrations of HbA1c and atherogenic lipids, as well as more severe hypoxemia compared to patients with normal FFA levels [23]. During CABG surgery, as a rule, the level of FFAs was not assessed before surgery; one of the studies showed that a postoperative increase in FFAs was associated with the development of hypoxemia and lung damage due to endothelial activation [24]. Indeed, it is known that FFAs are associated with a decrease in insulin sensitivity (tissue and hepatic) and an impaired carbohydrate metabolism due to the inhibition of glucose oxidation and stimulation of protein kinase C [25]. Even at the same baseline glucose levels, patients with high FFA levels during their stay in the intensive care unit had higher blood glucose levels and required higher doses of insulin than patients with normal free fatty acid levels. This also suggested an association between FFAs and insulin resistance in these patients.

According to a recent meta-analysis [26], it has been shown that in patients with DM, incomplete compensation of hyperglycemia, which is manifested by an increase in the level of HbA1c before surgery, is associated with an increased risk of infections in the area of the surgical wound and the development of renal failure and myocardial infarction during CABG. These data are quite expected. More interesting is the finding that in patients without DM, high preoperative HbA1c levels increase the risk of mortality and renal failure after CABG. Even more impressive results are presented by Tennyson et al. [27]: with an HbA1c level of >8.6%, the risk of death from CABG increased fourfold. However, not everything is so clear. Even in the mentioned meta-analysis in patients with DM, the number of strokes, deaths and length of stay in the ICU after CABG did not depend on the level of HbA1c. For patients without DM, there were no differences in the incidence of myocardial infarction and atrial fibrillation in patients with different HbA1c levels [26]. In addition, a number of other reviews have shown that elevated preoperative HbA1c levels do not increase postoperative morbidity and mortality in diabetic patients undergoing heart surgery [28,29]. Therefore, the researchers suggest conducting high-quality RCTs in the future with a large enough sample size to further study this issue. Our results are consistent with the conclusions of the meta-analysis—in the combined endpoint group after CABG, there was a higher level of HbA1c before surgery, but this indicator did not have an independent association with the studied endpoints.

The association of FFA levels before CABG with the development of a combined endpoint that we have revealed may have a specific clinical significance, since this level can be influenced during preoperative preparation of patients. For example, a study by Hosny et al. [30] showed that preoperative nocturnal lipid emulsion infusion resulted in significantly lower pre-induction FFAs, lower blood glucose and insulin levels, and lower postoperative serum TG and very-low-density lipoprotein levels compared to conventional preoperative fasting. This was also accompanied by a reduced need for postoperative inotropic support in the lipid infusion group. This study was performed in obese patients, and it is desirable to study this method of preoperative preparation in the general cohort of patients undergoing CABG.

### Study Limitation

There are some limitations of this study. First, we calculated several indices of insulin resistance, including fasting insulin and blood glucose levels, which could be affected by the glucose-lowering drugs taken by patients with type 2 diabetes. This must be taken into account in the general perception of the results, but at the same time, the calculation of insulin resistance indices in this category of patients for scientific purposes is legitimate and is used in scientific research. Secondly, we did not take into account the achievement of glycemic control goals in patients with diabetes before surgery. Third, in our study, we did not conduct a comprehensive troponin measurement after surgery; it was determined only in those patients who had symptoms of an ischemic event. Continuous determination of troponin would allow us to identify asymptomatic myocardial ischemia after surgery and more accurately determine the association of markers of insulin resistance with the development of ischemic events. Another limitation of the study is the fact that we did not calculate the sample size. This is because no pilot studies have been conducted on this topic before.

## 5. Conclusions

After CABG, in patients who developed perioperative complications and/or had a prolonged stay in the hospital compared to patients without complications, disorders of carbohydrate metabolism in the preoperative period were more often detected.

They were more likely to have diabetes mellitus and other disorders of carbohydrate metabolism, as well as a decreased QUICKI and an increased concentration of free fatty acids. Diabetes mellitus was one of the independent predictors of a length of stay in the hospital for more than 14 days. Among the independent predictors of the presence of a combined endpoint (serious postoperative complications + duration of hospitalization more than 10 days) were gender, age, and size of the left atrium, in addition to the level of free fatty acids before surgery.

## Figures and Tables

**Table 1:** Anamnestic and clinical characteristics of patients in groups with a combined endpoint and without it.

	Group 1 with Combined Endpoint *n* = 291	Group 2 without Combined Endpoint *n* = 92	*p*
Men/women (*n*, %)	205/86 (70.5/29.5)	79/13 (85.5/14.1)	0.003
Age (years, Me [LQ; UQ])	60.0 [55.0; 60.0]	57.0 [51.0; 60.5]	<0.001
Any disorders of carbohydrate metabolism (*n*, %)	156 (53.6)	36 (39.1)	0.015
Type 2 diabetes (*n*, %)	109 (37.5)	16 (17.4)	<0.001
Prediabetes (IFG, IGT) (*n*, %)	47 (16.2)	20 (21.7)	0.002
Normoglycemia (*n*, %)	135 (46.4)	56 (60.9)	0.002
Body mass index (kg/m^2^, Me [LQ; UQ])	29.1 [25.9; 32.2]	27.0 [24.1; 30.0]	<0.001
Obesity (*n*, %)	125 (42.9)	24 (26.1)	<0.001
Arterial hypertension (*n*, %)	264 (90.7)	79 (85.9)	0.185
Angina class III–IV (*n*, %)	113 (38.8)	34 (37.0)	0.591
Heart failure class NYHA III (*n*, %)	84 (28.9)	18 (19.6)	0.172
Arrhythmias (*n*, %)	112 (38.5)	17 (18.5)	0.502
Intermittent claudication (*n*, %)	49 (16.8)	14 (15.2)	0.714
Smoking (*n*, %)	86 (29.6)	42 (45.7)	0.004
Myocardial infarction history (*n*, %)	182 (62.5)	58 (63.0)	0.668
Stroke history (*n*, %)	21 (7.2)	6 (6.5)	0.820
Previous PCI (*n*, %)	26 (8.9)	11 (12.0)	0.392
Previous CABG (*n*, %)	82 (0.7)	2 (2.2)	0.797
Previous carotid surgery (*n*, %)	8 (2.8)	3 (3.3)	0.803
Previous lower limb artery surgery or amputation (*n*, %)	2 (0.7)	1 (1.1)	0.704
CABG characteristics			
Cardiopulmonary bypass (*n*, %)	268 (92.1)	78 (84.8)	0.038
Isolated coronary artery bypass grafting (*n*, %)	263 (90.4)	91 (98.9)	0.007
Combined surgery (*n*, %)	28 (9.6)	1 (1.1)	0.007
Aortic valve (*n*, %)	3 (1.0)	0 (0)	0.328
Mitral valve (*n*, %)	1 (0.3)	0 (0)	0.573
Carotid endarterectomy (*n*, %)	8 (8.2)	0 (0)	0.108
Ventriculoplasty (*n*, %)	13 (4.5)	1 (1.1)	0.132
Radiofrequency ablation (*n*, %)	14 (4.8)	0 (0)	0.032
Total duration of surgery (minutes, Me [LQ; UQ])	246.0 [210.0; 298.0]	210.0 [195.0; 264.0]	0.006
CPB duration (minutes, Me [LQ; UQ])	98.0 [81.0; 116.0]	86.5 [73.0; 103.0]	0.002
Aortic clamping time (minutes, Me [LQ; UQ])	63.0 [50.0; 75.0]	60.0 [49.0; 72.0]	0.331
Euro SCORE II (%, Me [LQ; UQ])	1.34 [1.23; 2.91]	1.32 [0.88; 2.10]	0.002
Hospital stay after CABG (days, Me [LQ;UQ])	13.0 [12.0; 17.0]	9.0 [8.0; 10.0]	0.003
Preoperative drugs			
Angiotensin II receptor antagonists (*n*, %)	11 (3.8)	5 (5.4)	0.480
Angiotensin-converting enzyme inhibitors (*n*, %)	250 (85.9)	81 (88.0)	0.602
Beta-blockers (*n*, %)	285 (97.9)	90 (97.8)	0.546
Potassium-sparing diuretics (*n*, %)	51 (17.5)	13 (14.1)	0.675
Thiazide-like diuretics (*n*, %)	31 (10.7)	7 (7.6)	0.451
Loop diuretics (*n*, %)	272 (93.5)	88 (95.7)	0.448
Calcium channel blockers (*n*, %)	195 (67.0)	55 (59.8)	0.204
Statins (*n*, %)	224 (77.0)	69 (75.0)	0.789
Metformin (*n*, %)	15 (5.2)	2 (2.2)	0.461
Sulfonylurea drugs (*n*, %)	42 (14.4)	3 (3.3)	0.004
Inhibitors DPP 4/GLP 1 receptor agonists (*n*, %)	7 (2.4)	1 (1.1)	0.724
Insulin therapy before hospitalization (*n*, %)	19 (9.9)	2 (2.2)	0.181
Insulin therapy during hospitalization (*n*, %)	49 (16.8)	4 (4.4)	0.002

**Notes:** IGT—impaired glucose tolerance, IFG—impaired fasting glucose, NYHA—New York Heart Association, PCI—percutaneous coronary intervention, CABG—coronary artery bypass grafting, CPB—cardiopulmonary bypass, EuroSCORE II—European System for Cardiac Operative Risk Evaluation, GLP 1—glucagon-like peptide 1, DPP 4—dipeptidyl peptidase 4.

**Table 2:** Preoperative laboratory and instrumental parameters in groups with a combined endpoint and without it.

	Group 1 with Combined Endpoint *n* = 291	Group 2 without Combined Endpoint *n* = 92	*p*
Creatinine (μmol/L)	83.5 [70.0; 103.0]	90.0 [73.0; 103.0]	0.165
GFR by CKD-EPI formula (mL/min/1.73 m^2^)	80.4 [64.0; 98.6]	81.4 [63.2; 98.6]	0.906
Glycated hemoglobin (HbA1c, %)	5.5 [5.1; 7.1]	5.3 [5.0; 5.6]	0.009
Glucose, venous plasma (mmol/L)	5.8 [5.2; 6.9]	5.5 [5.1; 6.2]	0.031
Triglycerides (mmol/L)	2.0 [1.4; 2.5]	1.6 [1.2; 2.2]	0.046
Insulin, IU/mL	10.1 [2.5; 23.5]	6.4 [2.6; 15,6]	0.418
HOMA-IR	2.52 [0.61; 6.26]	1.43 [0.74; 3.73]	0.083
QUICKI	0.14 [0.13; 0.18]	0.16 [0.14; 0.17]	0.136
FFA, mmol/L	0.4 [0.3; 0.6]	0.3 [0.2; 0.5]	0.007
Revised-QUICKI	0.17 [0.15; 0.22]	0.19 [0.16; 0.23]	0.020
McAuley IR index	6.24 [4.72; 8.98]	6.65 [5.55; 9.48]	0.110
Coronary angiography data			
1-vessel disease *	64 (22.0)	16 (17.4)	0.343
2-vessel disease *	88 (30.2)	25 (27.2)	0.274
3-vessel disease *	121 (41.6)	45 (48.9)	0.216
Left main coronary artery stenosis > 50%	61 (21.0)	24 (26.1)	0.302
Echocardiography data before surgery (Me [LQ; UQ])			
LV end-systolic volume (mL)	63.0 [47.0; 97.0]	62.2 [48.0; 96.5]	0.421
LV end-diastolic volume (mL)	156.0 [132.0; 191.0]	150.0 [129.0; 172.0]	0.125
LV end-systolic size (cm)	3.8 [3.4; 4.7]	3.8 [3.3; 4.6]	0.467
LV end-diastolic size (cm)	5.6 [5.2; 6.2]	5.5 [5.1; 6.0]	0.051
Left atrium (cm)	4.3 [4.0; 4.5]	4.2 [3.8; 4.4]	<0.001
LV ejection fraction (%)	60.0 [50.0; 64.0]	61.0 [50.0; 64.0]	0.958
LV myocardial mass by Deveraux and Reichek (g)	304.3 [250.5; 375.0]	242.1 [276.0; 333.7]	0.010
LV myocardial mass index (g/m^2^)	159.2 [133.5; 192.0]	150.3 [124.2; 175.0]	0.015

**Notes:** Me [LQ; UQ]—median with upper and lower quartile, GFR—glomerular filtration rate, CKD-EPI—Chronic Kidney Disease Epidemiology Collaboration, HOMA-IR—Homeostasis Model Assessment Of Insulin Resistance, QUICKI—Quantitative Insulin Sensitivity Check Index, FFAs—free fatty acids, LV—left ventricle, *—the number of involved main coronary arteries.

**Table 3:** Predictors’ complications or long hospitalizations after CABG (binary logistic regression analysis, Forward LR method).

Significant Complications							
	Predictors	B	SE	Wald	df	Sig.	Exp (B)
Step 1	Combined operations	1.421	0.574	6.121	1	0.013	4.143
	Constant	−1.421	0.211	45.570	1	0.000	0.241
**Hospital stay after CABG > 10 days**							
Step 1	Left atrium	2.304	0.555	17.246	1	0.000	10.017
	Constant	−8.170	2.212	13.637	1	0.000	0.000
Step 2	Male gender	−1.673	0.597	7.851	1	0.005	0.188
	Left atrium	2.474	0.578	18.347	1	0.000	11.870
	Constant	−7.555	2.296	10.826	1	0.001	0.001
Step 3	Male gender	−1.982	0.625	10.041	1	0.002	0.138
	Left atrium	2.096	0.601	12.159	1	0.000	8.130
	LV end-diastolic volume	0.671	0.348	3.710	1	0.054	1.956
	Constant	−9.535	2.602	13.430	1	0.000	0.000
**Hospital stay after CABG > 14 days**							
	Predictors	B	SE	Wald	df	Sig.	Exp (B)
Step 1	Type 2 diabetes	1.196	0.369	10.519	1	0.001	3.305
	Constant	−2.706	0.593	20.845	1	0.000	0.067
Step 2	Male gender	−0.984	0.395	6.191	1	0.013	0.374
	Type 2 diabetes	1.148	0.377	9.290	1	0.002	3.152
	Constant	−1.961	0.656	8.932	1	0.003	0.141
Step 3	Male gender	−1.142	0.408	7.821	1	0.005	0.319
	Left atrium	0.531	0.280	3.607	1	0.058	1.700
	Type 2 diabetes	1.070	0.383	7.793	1	0.005	2.916
	Constant	−4.026	1.283	9.849	1	0.002	0.018
**Combined end point** (**significant complications or hospital stay after CABG > 10 days**)							
	Predictors	B	SE	Wald	df	Sig.	Exp (B)
Step 1	Left atrium	2.060	0.544	14.353	1	0.000	7.844
	Constant	−7.070	2.169	10.623	1	0.001	0.001
Step 2	Male gender	−1.468	0.592	6.156	1	0.013	0.230
	Left atrium	2.176	0.556	15.310	1	0.000	8.807
	Constant	−6.399	2.226	8.261	1	0.004	0.002
Step 3	Male gender	−1.505	0.608	6.131	1	0.013	0.222
	Left atrium	2.347	0.588	15.924	1	0.000	10.452
	Free fatty acids	2.691	1.103	5.952	1	0.015	14.749
	Constant	−8.130	2.439	11.105	1	0.001	0.000
Step 4	Male gender	−1.309	0.623	4.418	1	0.036	0.270
	Age	0.069	0.035	3.913	1	0.048	1.072
	Left atrium	2.195	0.596	13.562	1	0.000	8.979
	Free fatty acids	2.559	1.088	5.532	1	0.019	12.917
	Constant	−11.640	3.080	14.282	1	0.000	0.000

**Notes:** B—coefficient for the constant in the null model, SE—standard error, Wald—Wald chi-square test that tests, df—degree of freedom for the Wald chi-square test, Exp (B)—exponentiation of the B coefficient, LV—left ventricle, CABG—coronary artery bypass grafting.

## Data Availability

The datasets used and/or analyzed during the current study available from the corresponding author on reasonable request.

## Supplementary Materials

The following supporting information can be downloaded at: https://www.mdpi.com/article/10.3390/biomedicines11112977/s1, Table S1: Model Summary for four different variants of binary logistic regression; Table S2: Omnibus Tests of Model Coefficients for four different variants of binary logistic regression; Table S3: Classification Table for four different variants of binary logistic regression.
