# Supplementary material for: The Role of Insulin Resistance in the Development of Complications after Coronary Artery Bypass Grafting in Patients with Coronary Artery Disease
Source: Biomedicines. 2023 Nov 5;11(11):2977. doi: 10.3390/biomedicines11112977 (PMC10669372; doi:10.3390/biomedicines11112977)
Supplement: Supplementary file 1 [file biomedicines-11-02977-s001.zip › biomedicines-2613176-supplementary.pdf]

Supplement

Suppl. Table S1 **Model Summary for four different variants of binary logistic regression**

step	-2 Log likelihood	Cox and Snell R Square	Nagelkerke R Square
**Significant Complications**			
1	161.278[a]	.036	.056
a. Estimation terminated at iteration number 4 because parameter estimates changed by less than .001.			
**Hospital stay after CABG >10 days**			
1	145.013[a]	.144	.219
2	134.927[a]	.197	.299
3	130.901[a]	.217	.330
a. Estimation terminated at iteration number 6 because parameter estimates changed by less than .001.			
**Hospital stay after CABG >14 days**			
1	176.018 [a]	.067	.096
2	169.882 [a]	.102	.147
3	166.037[b]	.124	.178
a. Estimation terminated at iteration number 4 because parameter estimates changed by less than .001.			
b. Estimation terminated at iteration number 5 because parameter estimates changed by less than .001.			
**Combined end point (significant complications or a hospital stay after CABG >10 days)**			
1	142.293[a]	.117	.182
2	134.596[a]	.159	.248
3	127.851[a]	.194	.303
4	123.596[a]	.215	.336
a. Estimation terminated at iteration number 6 because parameter estimates changed by less than .001.			

Suppl. Table S2. **Omnibus Tests of Model Coefficients for four different variants of binary logistic regression**

		Chi-square	df	Sig.
**Significant complications**				
Step 1	step	5,830	1	.016
	block	5,830	1	.016
	Model	5,830	1	.016
**Hospital stay after CABG >10 days**				
Step 3	step	4.026	1	.045
	block	38.685	3	.000
	Model	38.685	3	.000
**Hospital stay after CABG >14 days**				
Step 3	step	3.844	1	.050
	block	20.881	3	.000
	Model	20.881	3	.000
**Combined endpoint (significant complications or a hospital stay after CABG >10 days)**				
Step 4	step	4.256	1	.039
	block	38.337	4	.000
	Model	38.337	4	.000

Suppl. Table S3. **Classification Table for four different variants of binary logistic regression**

	Observed		Predicted		
			Significant complications		Percentage Correction
			0	1	
Step 1	Significant Complications	0	123	0	100.0
		1	35	0	.0
	overall Percentage				77.8
			Hospital stay after CABG >10 days		
Step 3	Hospital stay after CABG >10 days	0	0	36	.0
		1	0	122	100.0
	overall Percentage				77.2
			Hospital stay after CABG >14 days		
Step3	Hospital stay after CABG >14 days	0	114	0	100.0
		1	44	0	.0
	overall Percentage				72.2
			Combined end point (significant complications or hospital stay after CABG >10 days)		
Step 4	Combined end point (significant complications or hospital stay after CABG >10 days)	0	10	23	30.3
		1	11	114	91.2
	overall Percentage				78.5
a. Constant is included in the model.					
b. The cut value is .500					
